# The Impact of Chronic Pain on Cognitive Function

**DOI:** 10.3390/brainsci15060559

**Published:** 2025-05-24

**Authors:** Milan Patel, Jamal Hasoon, Rodrigo Diez Tafur, Giuliano Lo Bianco, Alaa Abd-Elsayed

**Affiliations:** 1 Department of Anesthesiology, School of Medicine and Public Health, University of Wisconsin, Madison, WI 53705, USA; mapatel5@wisc.edu; 2 Department of Anesthesiology, Critical Care, and Pain Medicine, University of Texas Health Science Center at Houston, Houston, TX 77030, USA; jamal.j.hasoon@uth.tmc.edu; 3 Centro MDRS: Sports, Spine & Pain Center, Pain Management Unit, Clinica Angloamericana, Lima 15073, Peru; rodrigo.dieztafur@mail.mcgill.ca; 4 Responsabile Unita’ Operativa Analgesia e Chirurgia Percutanea, Fondazione Istituto G. Giglio, 90015 Cefalù, Italy; giulianolobianco@gmail.com

**Keywords:** chronic pain, cognitive dysfunction, neuroinflammation, gray matter atrophy, depression, Alzheimer’s disease, Parkinson’s disease, pain interference, neurodegeneration, microglial activation, central sensitization, cognitive decline

## Abstract

**Background**: Chronic pain affects a significant proportion of the population in the United States and poses a significant public health concern. Beyond physical discomfort, chronic pain has been increasingly linked to cognitive dysfunction, including impairments in memory, attention, executive function, and decision-making. The relationship is particularly pronounced in older adults and may contribute to the onset or progression of neurodegenerative diseases. **Objective**: This comprehensive review explores the relationship between chronic pain and cognitive function, emphasizing the underlying neurobiological mechanisms, structural brain changes, and associated comorbidities. **Methods**: A review was conducted using peer-reviewed studies that began with the earliest pain and cognition mechanisms, followed by further investigation of cognitive effects of chronic pain, neuroimaging findings, and comorbid neuropsychiatric and neurodegenerative conditions. Sources included large-scale cohort studies, functional MRI analyses, and neurobiological investigations focusing on prefrontal cortex activity, default mode network alterations, and gray matter atrophy. **Results**: Chronic pain is associated with cognitive deficits through multiple converging pathways. It contributes to measurable impairments in cognitive function and is linked to structural and functional brain alterations. Regions of interest include the dorsolateral prefrontal cortex, medial prefrontal cortex, and default mode network, which can be connected to the neural resource hypothesis because of their cognitive domain impairments. A better understanding of these mechanisms highlights the importance of early recognition and multidisciplinary management strategies, including neuromodulation and cognitive rehabilitation. Future research should prioritize longitudinal studies and integrated interventions targeting both pain and cognitive health.

## 1. Introduction

In a 2019 National Health Interview Survey (NHIS) edition, approximately 50.2 million adults reported pain. This represents roughly 20.5% of the population reporting pain on most or all days. Alarmingly, this results in 1 in 5 adults in the United States reporting that pain is affecting their daily lives [1].

The NHIS defines chronic pain by asking individuals two questions: “In the past 3 months, how often did you have pain, would you never say, some days, most days, or every day?” and “Over the past 3 months, how often did pain limit your life or work activities, would you say never, some days, most days, or every day” [2]. Based on the results of these questions, NHIS personnel could further quantify the prevalence of chronic pain in that population.

A cohort study by Nahin et al. [2] was conducted to determine the significance of chronic pain within a large population. Using NHIS data from 2019–2020, it was found that in the sample of 10,415 participants, the results were relevant in showing chronic pain as a growing issue. At the conclusion of the study in 2020, it was found that there were 52.4 cases per 1000 person-years (PY) of chronic pain. Furthermore, high-impact chronic pain was observed at a rate of 12.0 cases per 1000 PY. Overall, the rate of chronic pain at a baseline standard across this study resulted in 462.0 cases per 1000 PY, highlighting the critical importance of addressing chronic pain and improving treatment strategies [2].

Within this comprehensive review, particular focus has been placed on the connection between chronic pain and cognitive decline. As previously shown, chronic pain is a growing condition in the United States, and its connection to cognitive function is of significance. It has been found that chronic pain and cognitive impairment are highly correlated in older adults. According to Chen et al., the relationship between chronic pain and cognitive decline is bidirectional [3]. It was reported that an estimated 50% of chronic pain patients also reported cognitive decline. This was found through an epidemiological analysis of community-dwelling people and pain clinics [3]. This comprehensive review will critically analyze the relationship between chronic pain and cognitive function. This review will analyze data from the past 25 years and investigate the current understanding and literature associated with chronic pain and cognitive function.

## 2. Pain and Cognitive Decline

Cognitive impairment can be described as a decline or negative impact in one or more cognitive domains, including memory, attention, numeracy, language, literacy, and orientation. When assessing the relationship between cognitive decline and aging, it can be observed that the rates of chronic pain influencing cognitive function have risen with increasing life expectancies. According to Chen et al., approximately 50 million individuals currently live with dementia, with projections estimating that this number will rise to 152 million by 2050 [3]. This growing burden highlights the importance of understanding how chronic pain may contribute to or exacerbate cognitive deterioration, particularly in aging populations.

The bidirectional relationship between chronic pain and cognitive decline is significant in understanding their relationship. Specifically, this review will highlight the effects of chronic pain in relation to the dorsolateral prefrontal cortex, medial prefrontal cortex, and default mode network and their effect on the cognitive domains of attention and memory. Chronic pain has shown a connection to cognitive decline through initial studies later discussed in this review; however, as mentioned earlier, this relationship is bidirectional. Cognitive decline can also have an impact on chronic pain, as potentially supported by ideas such as the neural resource hypothesis. Neural and nociceptive pathways flooding the body with a multitude of stimuli have been shown to lead to enhanced stress on the bodies of common chronic pain candidates, typically of the geriatric population [4]. Through many studies assessing neurological function and imaging, this relationship will be further explored throughout the contents of this review.

## 3. Neurobiological Mechanisms

### 3.1. Neural Resource Hypothesis

Chronic pain can be connected to aging and decreased quality of life (QoL) through the neural resource hypothesis. This hypothesis is grounded in the foundation that the persistence of chronic pain diverts the ability of certain regions of the brain to perform other tasks. Thus, the constant need for pain processing leads to the brain’s inability to process and execute different cognitive functions [5]. Grounds of support for the neural resource hypothesis stem from studies displaying reduced activity in the dlPFC and increased activity in the mPFC and DMN regions of the brain. Furthermore, neurological imaging-based studies have found enhanced grey matter atrophy in regions including prefrontal, temporal, cingulate, and somatomotor regions. In a study by Berryman et al., clinical observation revealed that many individuals reported poor memory and concentration [6]. The authors suggested that attentional resources are disproportionately directed toward the internal experience of pain, thereby limiting attention available for other cognitive processes.

Berryman et al. [6] made four statements throughout their study. Firstly, regarding the neural resource hypothesis, which has been mentioned previously. Secondly, the fact that bodily sensations can require more attention for chronic pain patients; this, in turn, diverts attention from other cognitive tasks. Repeated processing of the same stimulus—whether relevant or not—can lead to more efficient internal processing, potentially skewing attention toward irrelevant stimuli. Lastly, chronic pain inhibits and affects cortical inhibitory mechanisms, leading to impedance of deactivation in specific areas of the brain [6,7].

In another study conducted by Zhou et al. that focused on the connection between chronic low back pain and cognitive decline, competition for neural processing ability in the dorsolateral prefrontal cortex (dlPFC) was found [8]. This can be attributed to initial MRI imaging showing greater blood-oxygen dependent levels (BOLD) signals in individuals experiencing chronic low back pain and cognitive decline. This further supports the neural resource hypothesis, as the DLPFC is responsible for the executive function of many cognitive domains, and the need for constant pain processing detracts from the ability to carry out said functions.

The neural resource hypothesis serves as a basis for initial reasoning and connection between chronic pain and cognitive decline.

### 3.2. Brain Regions Implicated in Chronic Pain-Induced Cognitive Dysfunction

Chronic pain in relation to specific brain areas remains an area of increased interest. In a review conducted by Zhou et al. [8], there were five potential mechanisms found that link chronic low back pain to cognitive function. The five mechanisms reported by Zhou et al. [8] include (1) altered activity in the cortex and neural networks, (2) grey matter atrophy, (3) microglial activation and neuroinflammation, (4) comorbidities associated with CLBP, and (5) gut microbiota dysbiosis [8].

Beginning with altered activity in the cortex and neural networks, chronic low back pain significantly affects the DLPFC, mPFC, and default mode network (DMN). Prior literature has concluded that DLPFC activity has been diminished while there has been overactivation of mPFC and DMN [8].

#### 3.2.1. Dorsolateral Prefrontal Cortex (DLPFC)

The DLPFC in individuals with chronic back pain has been thought to be a central part of pain processing, as well as having many cognitive functions. According to Zhou et al., in theory, the DLPFC obeys the neural resource hypothesis, and individuals performing cognitive tasks with chronic low back pain (CLBP) would show less DLPFC activity [8]. However, a study conducted by Mao et al. [9] states the opposite. Individuals with CLBP displayed significantly lower DLPFC activity than those without CLBP during the Multi-Source Interference Task, a validated attention task for activating DLPFC [9].

Additionally, Baliki et al. [10] further demonstrate that individuals with higher-intensity CLBP have also shown increased resting-state BOLD signals in the mPFC and the rostral portion of the anterior cingulate cortex. These findings challenge the neural resource hypothesis. Baliki et al. state that there is an inverse relationship between the DLPFC and mPFC in BOLD signal intensity when individuals with CLPB perform complex cognitive tasks [10].

#### 3.2.2. Medial Prefrontal Cortex (mPFC)

Similar to the DLPFC, as previously mentioned, the mPFC is also a region of the brain of particular interest. In a study conducted by Hashmi et al., individuals with CLBP displayed significantly greater resting-state BOLD signals in the mPFC compared to individuals with subacute lower back pain [11]. According to Hashmi et al. and Baliki et al., it is noteworthy to acknowledge that individuals with CLBP were found to show mPFC overactivity when at a resting state [10,11]. This could be partly due to the need for the mPFC to process pain, leading to overactivity in this region.

With the mPFC, Zhou et al. [8] further explain a link between this region and pain catastrophizing. Pain catastrophizing is pain in relation to fear-inducing stimuli, which could play a role in the pain processing capability of the mPFC region [8]. In a key study conducted by Gracely et al., it was found that individuals with fibromyalgia with higher levels of pain catastrophizing indeed had significantly greater activity in the mPFC region [12]. This theory requires much further research and testing. However, initial results have suggested that pain catastrophizing may be linked to increased mPFC activity and overall overactivation in individuals with chronic pain.

#### 3.2.3. DLPFC and mPFC Connection

Increased mPFC activity is thought to be directly correlated to, or at least have some relationship with, DLPFC activity. As previously mentioned, these two regions are inversely proportional regarding their activity levels when assessing individuals with CLBP. Further comparisons can be made utlizing the sources shown in Table 1. However, this relationship could also hint at the effect of behind-the-scenes neural networks [8]. In a study by Kelly et al. [13], the BOLD signals of the default mode network (DMN) and dorsal attention network in healthy adults were strongly negatively correlated. This showed that the connection between these networks displayed better-sustained attention and decreased reaction time variation amongst the sample population [13].

Further research is needed to support the claims in these initial studies examining the relationship between DLPFC and mPFC. Their effect on cognitive domains such as memory and decision-making using BOLD signal analysis provides interesting results that warrant further exploration. Individuals with CLBP should be further assessed on a larger scale, and more studies should be conducted in which BOLD signal analysis regarding activity in the DLPFC and mPFC regions can be monitored and compared. The inverse relationship between these regions can serve as an excellent avenue for keying in specific experimental designs as further research is published.

## 4. Default Mode Network (DMN)

The DMN is a large neural network affecting cognitive performance in individuals with chronic pain. According to Zhou et al. [8], increased activation of the DMC, mPFC, and angular gyrus is associated with individuals exhibiting CLBP. The DMN has also been associated with the neural resource hypothesis. As mentioned in the study conducted by Baliki et al. [10], the potential cause of increased DMN activation could be this process. The need for pain processing in the DMN could be due to CLBP, which then affects the mPFC, in which activity is lessened [10].

Additionally, emotions consistent with depression and anxiety can also be involved. This has also been explored to answer why there is less deactivation in the DMN region of individuals with CLBP. In a study conducted by Simpson et al. [14], cerebral blood flow in the specific areas of the DMN was positively related to anxiety levels in the studied individuals. Upon being told that the participants would be receiving a painful electrical stimulation, the anxiety levels within these individuals rose, and thus, their DMN activity levels spiked [14]. In addition to the mPFC and DLPFC inverse relationship, the role of depression and anxiety as contributors to increased DMN activity should also be a point of further research.

Less deactivation of DMN is also linked to the cognitive domain of working memory. This results from the link between the lower deactivation of DMN and the decreased function of working memory. Although current research is lacking in this area, in individuals with CLBP, the lower deactivation of DMN in relation to this connection should be further investigated. This research would be crucial to explain further the intricate interaction between the DMN and mPFC regions and how cognitive domains are affected in individuals with CLBP. 

## 5. Psychological Theories of Pain and Cognition

Psychological theories offer additional insight into the relationship between chronic pain and cognitive dysfunction. This review will critically analyze two theories in particular: the Gate Control Theory of Pain and the Cognitive Load Theory.

### 5.1. Gate Control Theory of Pain Proposed by Melzack and Wall

Formulated in 1965, the Gate Control Theory states that the perception of pain is more than just nociceptive stimuli; it is also modulated by a “gate” mechanism. This occurs in the spinal cord, which also integrates sensory input and descending signals from the brain [21]. Psychological factors, including attention, emotion, and previous experiences, can influence this process. In individuals experiencing chronic pain, the gate can remain open longer or more frequently. Therefore, this can amplify pain signals and increase the cognitive burden on the brain.

This theory aligns with the findings of increased mPFC activity and decreased DLPFC activity, as emotional distress and attentional focus on pain can dysregulate the descending inhibitory pathways, leading to sustained cognitive engagement with pain-related stimuli.

### 5.2. Cognitive Load Theory (CLT)

The Cognitive Load Theory was initially developed to explain how the cognitive system in humans has limited capacity for processing information [22]. Additionally, chronic pain can occupy a large portion of working memory and other attentional resources. Thus, this results in reduced capacity for other cognitive tasks such as memory consolidation and executive function.

In situations where the brain is constantly utilizing resources for pain processing, fewer neurons and signals are able to be allocated for non-pain-related cognition. This overload, consistent with the neural resource hypothesis, may account for the impairments in working memory and executive function. This has been observed in multiple studies mentioned throughout this review and further supports the ground for the neural resource hypothesis and its connection to cognitive load theory.

### 5.3. Integration with Neurobiological Findings

The two theories just mentioned complement the findings presented in this review. The Gate Control Theory solidifies top-down processing and modulation of pain signals, which can be related to the changes seen in the mPFC and DMN activity. These affected areas include emotion and attention processing [11,23]. The Cognitive Load Theory, similarly, supports the neural resource hypothesis by showing how chronic pain conditions take up neurons from the brain, in which other tasks are now impaired. This leads to impaired function in the DLPFC in the form of executive functions [7].

## 6. Effect on Neurodegenerative Diseases and Comorbidities

### 6.1. Depression

In the current literature and clinical setting, cognitive impairment through chronic pain has been linked to depression. One study conducted by Sundermann et al. [24] compared similarly aged older individuals, with one group representing the control and the other group containing individuals with depression. cognitively normal older people to older individuals without depression. The study concluded that those with depression symptoms of a mild variety had approximately double the chance of mild cognitive impairment. This result is from a 4-year follow-up [24].

In a study conducted by Iwabuchi et al. [25], a large-scale fMRI study showed that out of the 225 individuals, a consensus was reached in which those with depression had increased regional homogeneity of voxel signals in the mPFC region. This is consistent with previous observations, which concluded that individuals with CLBP have elevated activity in the mPFC area of their brains [25]. Depression and comorbidities may play an increasing role in their connection to chronic pain. The symptoms and persistent pain may cause physiological changes within the brain, which should be explored further.

In the studies conducted by Corti et al. [26] and Tamburin et al. [27], the researchers sought to further investigate the potential connection between depression and chronic pain. Although the studies yielded results in which depression was not significantly related to cognitive performance in chronic pain individuals, the nature of the design and the small sample size suggest that these results should be taken cautiously [26,27]. Further research should be conducted to determine whether individuals with CLBP are at a greater risk of depression and decreased cognitive performance as it relates to decision-making. Based on the Iwabuchi study, there is certainly potential for a strong connection; however, larger studies should be conducted to confirm and further support this initial finding.

### 6.2. Alzheimer’s Disease

Alzheimer’s disease is a neurodegenerative disease that significantly impacts cognitive function. While, to this point, it has been discussed how chronic pain influences cognitive decline in individuals, this section will explore how persistent pain may affect the development of Alzheimer’s disease. In a study by Katz et al. [15], 1114 elderly patients were monitored and tracked. It was noted that over 4.4 years of tracking, 114 patients developed dementia [15,16]. This result is noteworthy as it further supports that higher pain interference levels were related to a higher risk of developing dementia. However, the intensity of the pain itself could not be directly tied to a greater risk of developing dementia.

In a study conducted by Ikram et al. [17], researchers sought to further build upon the connection previously stated, which is that pain interference levels can be related to Alzheimer’s disease and cognitive decline. In this study, Ikram et al. [17] aimed to find a relationship between osteoarthritis and pain interference. This study was carried out in a cross-sectional manner. The experimental groups were divided into three categories: osteoarthritis with pain interference with everyday activities, osteoarthritis without pain interference during normal activities, and no osteoarthritis with pain interference during everyday activities. The reference group included those who did not have osteoarthritis or pain interference. Overall, those with pain interference, regardless of whether they have osteoarthritis or not, were at greater risk of developing dementia or Alzheimer’s disease-related dementia (ADRD) [17].

Of significance, it cannot be stated that the intensity of pain has an impact on the risk of developing Alzheimer’s disease or ADRD. This could be a result of many different factors, including, but not limited to, underreporting of pain scores or cognitive impairment being so severe that there is difficulty in articulating an accurate pain score. Further research should be conducted to study the effect of pain intensity on the development of Alzheimer’s disease or ADRD, as the chronic pain population is in the higher age range and, thus, is already more susceptible to neurodegenerative diseases.

### 6.3. Parkinson’s Disease

In addition to depression and Alzheimer’s disease, Parkinson’s disease (PD) is also an area of growing research interest when assessing cognitive decline in relation to chronic pain. A study conducted by Vazririan et al. [28] sought to investigate whether chronic pain places individuals at a greater risk of developing Parkinson’s disease. The researchers identified three main categories to assess: PD, multiple system atrophy (MSA), and progressive supranuclear palsy (PSP). This study evaluated a large sample of 355,890 participants who did not qualify for any Parkinsonism categories mentioned earlier at baseline. The participants themselves were then placed into four groups: no chronic pain, having one or two pain sites, having three or four pain sites, and pain “all over the body”. The pain sites included the hip, neck/shoulder, back, and knee. The results were obtained and analyzed using a multivariable-adjusted Cox regression. Overall, the results showed that at a median follow-up time of 13.0 years, 2044 patients developed PD.

Furthermore, 77 individuals developed MSA, and 126 participants developed PSP. Using these data then for the multivariable analysis, it was found that individuals with pain at three and four or more sites had an 11% and 49% chance, respectively, of increased risk of getting PD [29]. This study is an introduction to chronic pain, further poking its head into another neurodegenerative disease. While the sample size of this study was significant, additional studies analyzing PD and chronic pain should be conducted to support this initial connection further.

Chronic pain and PD, on their own, have been associated with neuroinflammation and dopaminergic pathway disruption. Elevated levels of pro-inflammatory cytokines (TNF-alpha and IL-6) have been found in chronic pain and PD [30,31]. Therefore, a shared neuroimmune pathway contributes to cognitive impairment and overall neurodegeneration. These immune-mediated mechanisms could accelerate neuronal dysfunction in brain areas responsible for both pain modulation and executive function, such as the basal ganglia, prefrontal cortex, and thalamus.

Functional neuroimaging studies in PD patients have often shown cortical thinning and abnormal connectivity in pain-related networks, particularly the insula and anterior cingulate cortex [32], regions also implicated in chronic pain. Furthermore, cognitive domains such as attention, planning, and visuospatial processing are further compromised in patients who also have chronic pain. This compounding effect may be partially medi4ated by the persistent activation of pain-processing regions, similarly to the neural resource hypothesis, and could reduce the availability of cognitive bandwidth for higher-order tasks [7].

The bidirectional nature of this relationship deserves attention. Chronic pain is not only a potential risk factor for developing PD, but PD itself is known to alter pain perception. Up to 85% of patients with Parkinson’s disease report chronic pain [33], and these symptoms often precede motor deficits [34]. This suggests that neurodegenerative processes involved in PD may impair endogenous pain inhibitory pathways early in disease progression.

Finally, dopaminergic medications used in PD treatment, such as levodopa, may influence both pain perception and cognitive function. This further complicates the interpretation of clinical symptoms. While dopamine replacement therapy can reduce certain types of pain [35], it may also contribute to cognitive fluctuations [36].

## 7. Gray Matter Changes in Chronic Pain and Cognitive Decline

The impact of chronic pain on cognitive function and possible impairment can be connected to specific physiological changes. One key factor studied is the change in gray matter volume. In neurodegenerative diseases, such as Alzheimer’s disease, gray matter volume abnormalities have been noticed in neurological imaging, indicating there may be a potential connection between gray matter volume and chronic pain in relation to neurodegeneration [15].

Individuals with CLBP have been shown to have reduced gray matter volume and have been studied in the past. In a study by Ng et al. [19], a systematic search of studies was conducted to analyze the relationship between gray matter and CLBP. Of the 55 studies that met the criteria, 10 out of 15 qualified structural MRI studies showed decreased gray matter. Also, seven out of eight qualified studies showed white matter changes when comparing the CLBP groups to controls [16]. In relation to some regions of the brain previously mentioned, there was also reduced gray matter in the DLPFC [17], mPFC [17], insula [18], posterior cingulate cortex, cuneus, thalamus, temporal lobes, and precentral/postcentral gyrus [8].

Specifically, the DLPFC and mPFC are discussed in this review, as they are imperative to understanding cognitive decline with chronic pain. In the study conducted by Fritz et al. [20], researchers sought to compare gray matter and chronic pain through analysis of these key brain regions. The researchers employed voxel-based morphometry to perform this analysis. Fritz et al. [20] analyzed the imaging of 111 individuals with chronic low back pain and 432 controls to carry out this study. Overall, it was found that chronic back pain was connected to decreased gray matter in the ventrolateral prefrontal cortex, dorsolateral prefrontal cortex, ventral medial prefrontal cortex, and dorsal medial prefrontal cortex.

Regarding pain intensity, there was a correlation between gray matter volume, albeit a weak one. This was present in the left dorsolateral prefrontal cortex and anterior cingulate cortex. Noteworthy, the chronic back pain group displayed alterations in the areas commonly associated with pain processing and emotional function, further supporting previous information in this review article [17].

Although the DLPFC and mPFC are key contributors to the working theory behind the connection of cognitive decline and chronic pain via gray matter atrophy, it is worth noting the significance that the hippocampus and amygdala play. In a study by Nickl-Jockschat et al. [37], a further meta-analysis was conducted to explore this connection. A total of 22 studies consisting of 917 individuals with MCI were analyzed. This study showed that within this population, the most significant reduction of gray matter occurred in the hippocampus and amygdala [19]. Furthermore, an additional study conducted by Driscoll et al. [38] also focused on this exact topic. Driscoll et al. [19] conducted a 10-year longitudinal follow-up study through the Baltimore Longitudinal Study of Aging. One hundred thirty-eight individuals were analyzed across this study annually for 10 years. Eighteen of the participants were diagnosed with MCI during this study, during which a mixed-effects regression was used to compare brain volumes across regions. Overall, the chosen regional portions of the brain all declined. In particular, the ventricular CSF, frontal gray matter, superior, middle, and medial frontal, and superior parietal regions were affected. In addition to changes in temporal gray matter, this experiment also showed changes in whole brain volume, orbitofrontal, and temporal association cortices [20]. Gray matter atrophy has shown promising results and should be further studied to better assess the onset of neurodegenerative diseases and potentially alternative treatment options.

## 8. Future Directions and Conclusions

This review highlights the need for further research and studies observing chronic pain. Chronic pain is a global issue with very high prevalence rates across the world, significantly affecting quality of life and healthcare systems. There is growing interest in learning about how chronic pain affects cognitive function, as many patients report difficulties with memory, attention, and executive functioning. This review acquired sources from within the past 25 years and is a comprehensive review of the current state of chronic pain in relation to cognitive capabilities, integrating both neuroimaging and theoretical frameworks.

Beginning with assessing key regional brain areas, the DLPFC, mPFC, and DMN were crucial in understanding the initial findings relating to cognitive function and chronic pain. These areas are heavily involved in executive functioning, emotional regulation, and internal mentation. The activity levels within the DLPFC and mPFC were significant, as these regions showcased an inverse relationship when compared against each other. This finding may indicate a compensatory mechanism or imbalance that develops over time. The neural resource hypothesis was significant as it served as the starting point for where initial theories and possible experiments came from. The relationship between different cognitive regions began with the neural resource hypothesis, as this was the initial explanation for the decline in function in chronic pain sufferers. Supplemented with gray matter atrophy, for example, magnetic resonance imaging and voxel-based studies further solidified changes occurring in the brain, leading to a potentially increased risk of depression and neurodegenerative diseases. These structural alterations may be long-term consequences of sustained nociceptive signaling and stress on the brain. With the pain processing pathways, the DMN played a significant role in understanding how working memory and additional cognitive domains were affected in individuals with chronic back pain. The DMN also affected the mPFC, which was observed in the studies mentioned at the beginning of the review, suggesting a dynamic interplay between resting state and active cognitive networks.

Certain structural aspects, such as microglial significance, which observed the immune system and gray matter atrophy for overall volume, were also key contributors. Microglial activation, often seen in chronic pain states, may mediate inflammation-driven cognition and brain volume changes. However, the greatest room for future research should be comorbidities and neurodegenerative diseases. Greater emphasis on observing chronic pain in relation to depression, Alzheimer’s disease, and Parkinson’s disease should be prioritized, as shared pathways and overlapping symptoms suggest possible bidirectional relationships. This could help identify biomarkers and shared mechanisms that explain cognitive decline across multiple conditions. With more research available in testing and further solidifying the initial results, alternative treatment options such as nerve stimulation or other emerging technologies could be in play. Specifically, transcranial magnetic stimulation, direct current stimulation, and vagus nerve stimulation could be interesting potential treatment options with promising safety profiles. Regarding repeated transcranial magnetic stimulation (rTMS), this method has seen some usage in treating spinal cord injury, stroke, multiple sclerosis (MS), and facial pain.

Furthermore, it has shown some improvements in motor function [37] and has the potential for cognitive enhancement in other populations. rTMS and tDCS have also shown signs of anti-nociceptive effects and pain reduction, blending in with the neural resource hypothesis [38] and providing a physiological basis for their efficacy. Findings such as this one should be further tested and continue to be observed with larger sample sizes and longitudinal designs. Future experiments could also look into the neural resource hypothesis by testing the stimulation of multiple processing pathways while monitoring brain activity. Functional imaging during stimulation could offer real-time insights into network-level changes. Understanding the regions of the brain expressing overactivation or lessened activation is significant for tailoring the following line of testing for a better understanding of this growing connection between chronic pain and cognitive function. Personalized approaches yield better outcomes than traditional one-size-fits-all treatments.

The possibilities for neuromodulation techniques can help improve CLBP patients facing steep cognitive declines. These interventions could eventually be combined with pharmacological therapies or cognitive training to create multimodal treatment strategies. Additional interdisciplinary collaboration between neuroscience, immunology, and pain management could yield more nuanced insights and practical applications [39,40]. Moreover, integrating machine learning approaches to analyze neuroimaging and cognitive data could provide predictive modeling capabilities for early intervention strategies and personalized medicine. Future clinical trials should strive to include cognitive outcomes as secondary endpoints to better capture the full impact of chronic pain. Overall, chronic pain affects mental function, as seen in the studies presented in this comprehensive review. However, there is significant room for improvement and further research as treatment options and our understanding continue to be tested and grow. As interest in this intersection expands, the potential for discoveries remains high.

## Figures and Tables

**Table 1:** Overall groupings of the key affected areas and structural components, with key sources listed.

Group	Term	Sources
Brain Region	Dorsolateral Prefrontal Cortex (DLPFC)	Zhou et al. (2022) [8] Mao et al. (2014) [9] Baliki et al. (2006) [10]
Brain Region	Medial Prefrontal Cortex (mPFC)	Zhou et al. (2022) [8] Mao et al. (2014) [9] Baliki et al. (2006) [10] Hashmi et al. (2013) [11] Gracely et al. (2004) [12]
Neural Network	Default Mode Network (DMN)	Baliki et al. (2006) [10] Simpson et al. (2001) [14]
Brain Structure	Gray Matter	Zhou et al. (2022) [8] Katz et al. (2012) [15] Ezzati et al. (2019) [16] Ikram et al. (2019) [17] Malfliet et al. (2017) [18] Ng et al. (2018) [19] Fritz et al. (2016) [20]
